# Characteristics of Pediatric In-Hospital Cardiac Arrests and Resuscitation Duration

**DOI:** 10.1001/jamanetworkopen.2024.24670

**Published:** 2024-07-30

**Authors:** Amanda O’Halloran, Ryan W. Morgan, Kevin Kennedy, Robert A. Berg, Cody-Aaron Gathers, Maryam Y. Naim, Vinay Nadkarni, Ron Reeder, Alexis Topjian, Heather Wolfe, Monica Kleinman, Paul S. Chan, Robert M. Sutton

**Affiliations:** 1 Department of Anesthesiology and Critical Care Medicine, University of Pennsylvania Perelman School of Medicine and Children’s Hospital of Philadelphia, Philadelphia; 2 Leonard Davis Institute of Health Economics, University of Pennsylvania, Philadelphia; 3 Saint Luke’s Mid America Heart Institute, Kansas City, Missouri; 4 Department of Pediatrics, University of Utah, Salt Lake City; 5 Department of Anesthesiology, Critical Care and Pain Medicine, Harvard Medical School and Boston Children’s Hospital, Boston, Massachusetts

## Abstract

**Importance:**

Cardiopulmonary resuscitation (CPR) duration is associated with cardiac arrest survival.

**Objectives:**

To describe characteristics associated with CPR duration among hospitalized children without return of circulation (ROC) (patient-level analysis) and determine whether hospital median CPR duration in patients without ROC is associated with survival (hospital-level analysis).

**Design, Setting, and Participants:**

This retrospective cohort study of patients undergoing pediatric in-hospital CPR between January 1, 2000, and December 31, 2021, used data from the Get With the Guidelines–Resuscitation registry. Children receiving chest compressions for at least 2 minutes and/or defibrillation were included in the patient-level analysis. For the hospital-level analysis, sites with at least 20 total events and at least 5 events without ROC were included. Data were analyzed from December 1, 2022, to November 15, 2023.

**Exposures:**

For the patient-level analysis, the exposure was CPR duration in patients without ROC. For the hospital-level analysis, the exposure was quartile of median CPR duration in events without ROC at each hospital.

**Main Outcomes and Measures:**

For the patient-level analysis, outcomes were patient and event factors, including race and ethnicity and event location; we used a multivariable hierarchical linear regression model to assess factors associated with CPR duration in patients without ROC. For the hospital-level analysis, the main outcome was survival to discharge among all site events; we used a random intercept multivariable hierarchical logistic regression model to examine the association between hospital quartile of CPR duration and survival to discharge.

**Results:**

Of 13 899 events, 3859 patients did not have ROC (median age, 7 months [IQR, 0 months to 7 years]; 2175 boys [56%]). Among event nonsurvivors, median CPR duration was longer in those with initial rhythms of bradycardia with poor perfusion (8.37 [95% CI, 5.70-11.03] minutes; *P* < .001), pulseless electrical activity (8.22 [95% CI, 5.44-11.00] minutes; *P* < .001), and pulseless ventricular tachycardia (6.17 [95% CI, 0.09-12.26] minutes; *P* = .047) (vs asystole). Shorter median CPR duration was associated with neonates compared with older children (−4.86 [95% CI, −8.88 to −0.84] minutes; *P* = .02), emergency department compared with pediatric intensive care unit location (−4.02 [95% CI, −7.48 to −0.57] minutes; *P* = .02), and members of racial or ethnic minority groups compared with White patients (−3.67 [95% CI, −6.18 to −1.17]; *P* = .004). Among all CPR events, the adjusted odds of survival to discharge differed based on hospital quartile of median CPR duration among events without ROC; compared with quartile 1 (15.0-25.9 minutes), the adjusted odds ratio for quartile 2 (26.0-29.4 minutes) was 1.22 (95% CI, 1.09-1.36; *P* < .001); for quartile 3 (29.5-32.9 minutes), 1.23 (95% CI, 1.08-1.39; *P* = .002); and for quartile 4 (33.0-53.0 minutes), 1.04 (95% CI, 0.91-1.19; *P* = .58).

**Conclusions and Relevance:**

In this retrospective cohort study of pediatric in-hospital CPR, several factors, including age and event location, were associated with CPR duration in event nonsurvivors. The odds of survival to discharge were lower for patients at hospitals with the shortest and longest median CPR durations among events without ROC. Further studies are needed to determine the optimal duration of CPR during pediatric in-hospital cardiac arrest and to provide training guidelines for resuscitation teams to eliminate disparities in resuscitation care.

## Key Points

**Question:**

What factors are associated with longer cardiopulmonary resuscitation (CPR) duration among children with in-hospital cardiac arrest without return of circulation (ROC)?

**Findings:**

In this cohort study examining pediatric in-hospital cardiac arrest in 3859 patients, several factors, including age and event location, were associated with duration of CPR attempts among children without ROC. The odds of survival to discharge after cardiac arrest were lowest at sites that performed the shortest and longest median CPR attempts in events without ROC.

**Meaning:**

Further investigation is needed to determine which children are likely to benefit from longer CPR attempts.

## Teaser

This cohort study evaluates patient and event characteristics associated with cardiopulmonary resuscitation (CPR) duration among pediatric patients with in-hospital cardiac arrest without return of circulation and whether hospitals with longer median durations of CPR have higher survival rates among all children requiring CPR.

## Introduction

In the US, 15 000 hospitalized children receive cardiopulmonary resuscitation (CPR) annually.^1^ Many of these children do not survive, and among survivors, morbidity is common.^2,3,4,5^ Studies suggest that CPR duration is associated with survival. While pediatric in-hospital cardiac arrest (IHCA) survival rates are lower among children requiring longer durations of CPR, survival rates exceed 10% even among those receiving prolonged CPR.^6^

Adult IHCA studies using CPR duration in events without return of spontaneous circulation (ROSC) as a proxy for resuscitation effort intensity^7,8^ have identified associations between CPR duration and patient characteristics and outcomes. Longer CPR attempts in adult IHCA without ROSC are associated with patients who are younger and female and have a shockable initial rhythm.^7^ Moreover, there is an association between hospitals that perform longer CPR in patients without ROSC and higher survival rates among all events at these hospitals.^8^ While the mechanism for this hospital-level association between CPR duration and survival is unknown, variable resuscitation systems of care may contribute. Given the known differences between pediatric and adult IHCA,^5^ whether these associations persist among children undergoing CPR is an important knowledge gap that can provide insight into potentially modifiable disparities in pediatric IHCA care.

To that end, we performed a pediatric IHCA study using CPR duration among event nonsurvivors (patients without return of circulation [ROC]) as a proxy for resuscitation effort intensity. Our objectives were to evaluate patient and event characteristics associated with CPR duration during events without ROC (patient-level analysis) and determine whether hospitals performing longer median durations of CPR in patients without ROC (ie, hospitals with higher resuscitation effort intensity) have higher survival rates among all children requiring CPR at their hospital (hospital-level analysis). We hypothesized that patient characteristics, including race and age, would be associated with CPR duration in patients without ROC and that patients receiving CPR at hospitals that provide longer durations of CPR would have higher survival rates compared with those treated at hospitals providing shorter durations of CPR.

## Methods

### Data Source

This retrospective cohort study used the Get With the Guidelines–Resuscitation (GWTG-R) registry, a large, prospective, North American IHCA quality improvement registry provided by the American Heart Association (AHA). The design has been described previously.^9^ Briefly, hospitals submit Utstein Resuscitation Registry–style data^10^ regarding medical history, hospital care, and patient outcomes using an online, interactive case report form and Patient Management Tool (IQVIA). IQVIA serves as the data collection (through their Patient Management Tool) and coordination center for the AHA–American Stroke Association GWTG programs.

All participating institutions were required to comply with local regulatory and privacy guidelines and, if required, to secure institutional review board approval. Because data were used primarily at the local site for quality improvement, sites were granted a waiver of informed consent under the Common Rule. The study was deemed non–human participants research by the Children’s Hospital of Philadelphia Institutional Review Board and thus exempt from approval. We followed the Strengthening the Reporting of Observational Studies in Epidemiology (STROBE) reporting guideline.

### Study Population

We included pediatric (aged <18 years) patients with an index CPR event enrolled in the GWTG-R registry between January 1, 2000, and December 31, 2021. To ensure patients were meant to undergo a sustained resuscitation, we restricted the cohort to patients with at least 2 minutes of chest compressions and/or defibrillation. Events occurring in newborns (newborn illness category and/or event location of delivery suite, newborn nursery, or neonatal intensive care unit [NICU]) were included. We excluded events with initial rhythms other than asystole, pulseless electrical activity (PEA), ventricular fibrillation, pulseless ventricular tachycardia, or bradycardia with poor perfusion. Events were also excluded if CPR duration, outcome, or important covariates were missing; illness category was obstetric or visitor; or event location was ambulatory, rehabilitation, or same-day surgical. Events beginning before hospital arrival were not included.

Race and ethnicity, which were self-reported, were important to consider in this study given the prevalent race and ethnicity–based differences in pediatric health care. Race was coded using the following categories: Asian, Black, White, and other or unknown (other included American Indian or Alaska Native and Native Hawaiian or Other Pacific Islander race [given small numbers] and a GWTG-designated other category). Patients’ neurologic status was measured using Pediatric Cerebral Performance Category (PCPC) scores, which range from 1 (normal) to 6 (death).^11^

### Study Variables

In the patient-level analysis evaluating factors associated with duration of resuscitative efforts among patients without ROC, the dependent variable was CPR duration. Duration of CPR was calculated as the time from the start of CPR until the time CPR was stopped due to termination of resuscitation. For the hospital-level analysis, the outcomes were survival to discharge (primary) and sustained ROSC (secondary).

### Statistical Analysis

#### Patient-Level Analysis

Baseline characteristics of patients without ROC were compared across quartiles of CPR duration using Kruskal-Wallis tests for continuous variables and χ^2^ tests for categorical variables. To assess factors associated with CPR duration in patients without ROC, we constructed a multivariable hierarchical linear regression model, with site as a random effect to account for patient clustering within the site. Duration of CPR, treated continuously and truncated at 120 minutes, was the dependent variable. Variables considered for inclusion as fixed effects were primarily based on those associated with cardiac arrest outcomes in previous studies,^12,13,14,15,16,17^ including year, age at the time of event (neonates [≤30 days], infants [31 days to <1 year], young children [1-8 years], and older children [>8 years]),^12^ sex, race and ethnicity, event location (pediatric ICU, emergency department, NICU, cardiac ICU, other ICU, operating room or procedural area, newborn area, and other inpatient), time of arrest (day vs night), initial rhythm (ventricular fibrillation, pulseless ventricular tachycardia, asystole, PEA, or bradycardia with poor perfusion), illness category (medical cardiac, medical noncardiac, surgical cardiac, surgical noncardiac, trauma, or newborn), application of an automated external defibrillator, and use of a hospital-wide response. In addition, the following conditions coded as present prior to cardiac arrest were evaluated for the model: congestive heart failure; kidney, hepatic, or respiratory insufficiency; baseline neurologic deficits (measured using the admission PCPC); acute stroke; acute nonstroke neurologic event; pneumonia; hypotension; arrhythmia; sepsis; major trauma; metabolic or electrolyte abnormality; and metastatic or hematologic malignant neoplasm. Finally, we considered for model inclusion several critical care interventions (mechanical ventilation, vasoactive medications) in place at the time of IHCA. Of these candidate factors, those with a univariable association (*P* < .05) with CPR duration were included in the model (eTable 1 in Supplement 1). Interaction testing was not performed as we did not have relevant prespecified hypotheses in this exploratory study.

#### Hospital-Level Analysis

To evaluate whether hospital resuscitation effort intensity (ie, median CPR duration in patients without ROC) was associated with pediatric in-hospital CPR outcomes, we considered all children receiving CPR who met our inclusion criteria. Events achieving ROC through extracorporeal CPR (ECPR) were included with patients who achieved ROSC. Because this was a site-level analysis, we restricted the cohort to hospitals with at least 20 events and a minimum of 5 events without ROC, as the latter was used to define hospital CPR duration. All included hospitals were divided into quartiles based on median CPR duration among events without ROC.

Baseline characteristics of all events across hospital quartiles of CPR duration for patients without ROC were evaluated using Kruskal-Wallis tests for continuous variables and χ^2^ tests for categorical variables. We constructed a random intercept multivariable hierarchical logistic regression model to examine the association between hospital quartile of CPR duration and survival to discharge, with hospital site modeled as a random effect. Variables considered for model inclusion were similar to those for the patient-level analysis. Since hospital characteristics may impact CPR duration, we compared teaching status, size, and geographic region across quartiles of site CPR duration.

Analyses were evaluated at a 2-sided significance level of *P* < .05 and were conducted using SAS, version 9.4 (SAS Institute Inc). Data were analyzed from December 1, 2022, to November 15, 2023.

## Results

### Patient-Level Analysis

**Figure 1.**
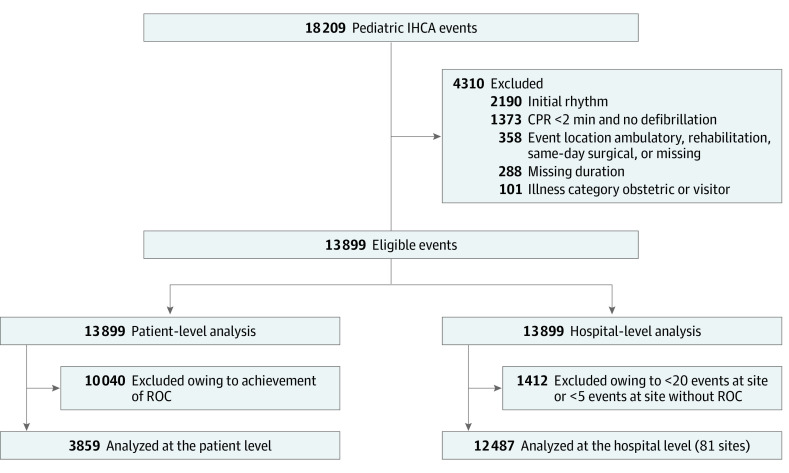
Study Flow Diagram CPR indicates cardiopulmonary resuscitation; IHCA, in-hospital cardiac arrest; and ROC, return of circulation.

**Figure 2.**
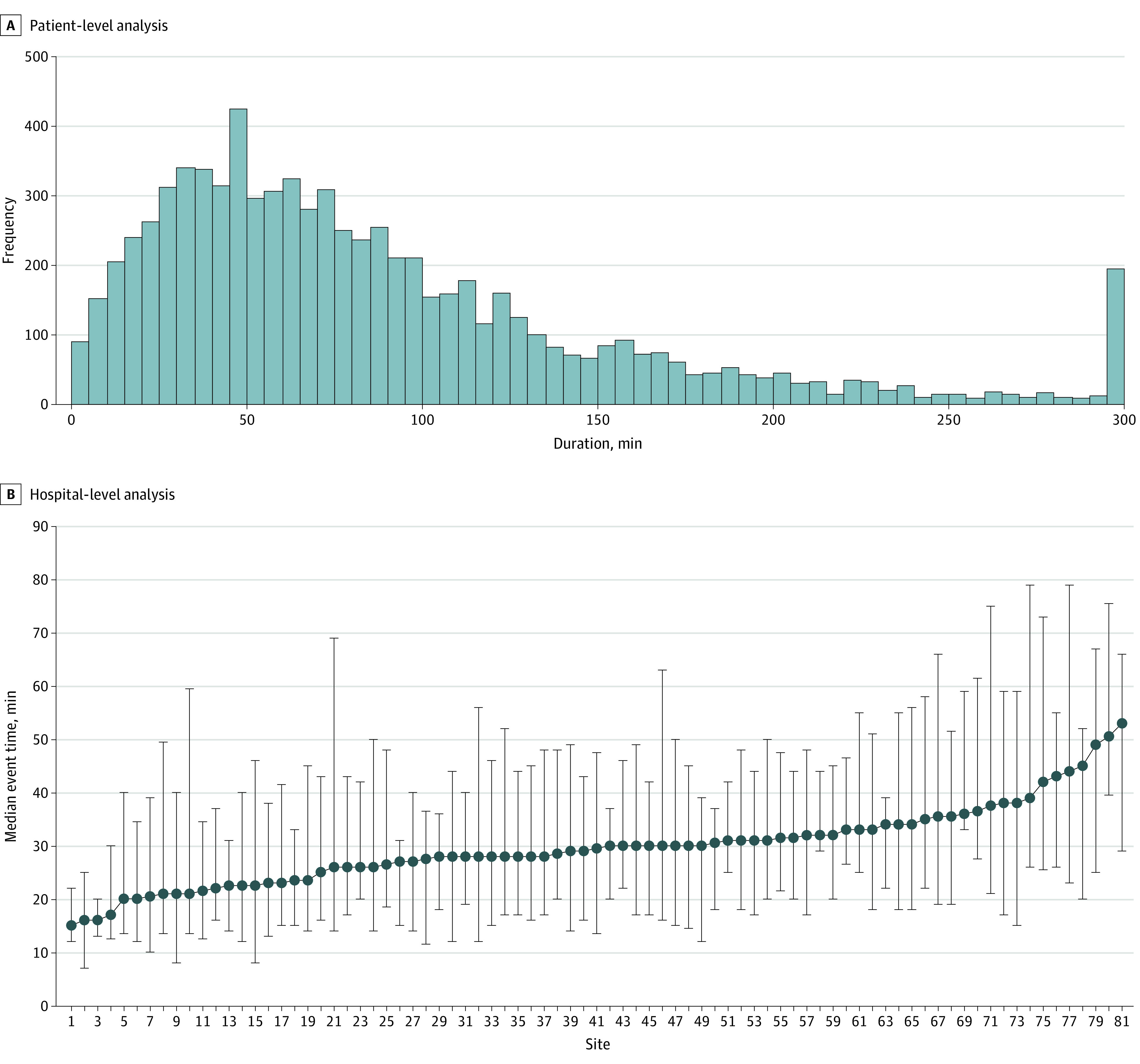
Distribution of Cardiopulmonary Resuscitation (CPR) Duration and Hospital Median CPR Duration in Events Without Return of Circulation Error bars indicate 95% CI.

Of 13 899 eligible events, 3859 events in patients without ROC comprised the patient-level cohort; this group included 1684 girls (44%) and 2175 boys (56%), with a median age of 7 months (IQR, 0 months to 7 years). In terms of race and ethnicity, 106 patients (3%) were Asian, 1070 (28%) were Black, 705 (18%) were Hispanic, 1888 (49%) were White, and 795 (21%) were of other or unknown race or ethnicity (Figure 1 and eTables 2 and 3 in Supplement 1). Among events without ROC, neonates were the most highly represented age group (1315 [34%]), and most neonatal events occurred in NICUs (884 of 1315 [67%]) (eTable 2 in Supplement 1). The median CPR duration was 28 (IQR, 16-46) minutes (Figure 2A). By contrast, among those with ROC, the median CPR duration was 7 (IQR, 3-20) minutes (eFigures 1 and 2 in Supplement 1).

**Figure 3.**
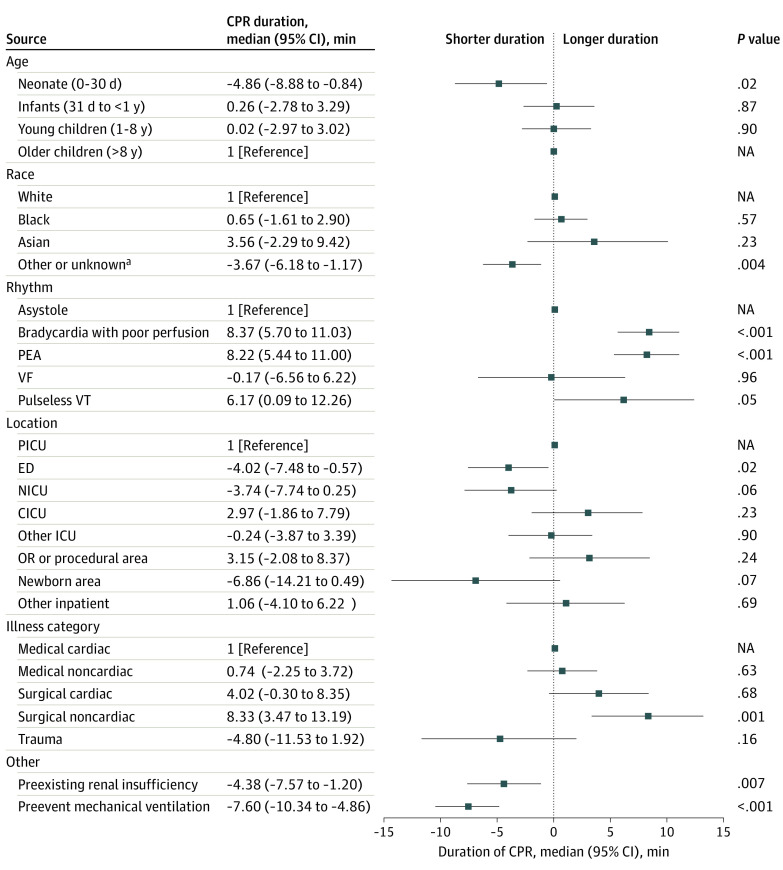
Multivariable Model of Patient-Level Analysis: Key Patient and Event Factors Associated With Cardiopulmonary Resuscitation (CPR) Duration in Events Without Return of Circulation CICU indicates cardiac intensive care unit (ICU); ED, emergency department; NA, not applicable; NICU, neonatal ICU; OR, operating room; PEA, pulseless electrical activity; PICU, pediatric ICU; VF, ventricular fibrillation; and VT, ventricular tachycardia. ^a^Includes self-reported American Indian or Alaska Native, Native Hawaiian or Other Pacific Islander, other, and unknown.

After multivariable adjustment, there was no association of median CPR duration with male sex (1.19 [95% CI, −0.72 to 3.10] minutes; *P* = .22) or with Black compared with White race (0.65 [95% CI, −1.61 to 2.90] minutes; *P* = .57) (eTable 4 in Supplement 1). Several characteristics were associated with CPR duration (Figure 3 and eFigure 3 in Supplement 1). Median duration of CPR was shorter in neonates compared with older children (−4.86 [95% CI, −8.88 to −0.84] minutes; *P* = .02), as were events among racial and ethnic minority patients compared with those in White patients (−3.67 [95% CI, −6.18 to −1.17] minutes; *P* = .004) and in patients with pre-event mechanical ventilation (−7.60 [95% CI, −10.34 to −4.86] minutes; *P* < .001) and kidney insufficiency (−4.38 [95% CI, −7.57 to −1.20] minutes; *P* = .007). Events in the emergency department received median shorter CPR duration than those in the pediatric ICU (−4.02 [95% CI, −7.48 to −0.57] minutes; *P* = .02). The median duration differed by initial rhythm: bradycardia with poor perfusion (8.37 [95% CI, 5.70-11.03] minutes; *P* < .001), PEA (8.22 [95% CI, 5.44-11.00] minutes; *P* < .001), and pulseless ventricular tachycardia (6.17 [95% CI, 0.09-12.26] minutes; *P* = .047) were associated with longer CPR durations compared with asystole.

### Hospital-Level Analysis

The cohort included 81 sites (Figure 1) with a median of 7.6 (IQR, 3.2-15.2) events per site per year and a median of 22 (IQR, 13-52) events without ROC per site contributing to hospital median CPR duration (Figure 2B). Sites were divided into quartiles based on median CPR duration in events without ROC, with quartile duration ranges as follows: 15.0 to 25.9 (quartile 1), 26.0 to 29.4 (quartile 2), 29.5 to 32.9 (quartile 3), and 33.0 to 53.0 (quartile 4) minutes.

Table 1 summarizes patient and event characteristics by hospital quartile of median CPR duration among patients without ROC. When stratifying events by patient race, only 372 of 3319 events (11%) occurring in Black children were at quartile 4 hospitals (vs 1327 of 6345 events [21%] occurring in White children). Children with an illness category of newborn were underrepresented at quartile 4 hospitals (129 of 1654 [8%] compared with 683 of 1978 [35%] for surgical cardiac and 316 of 1115 [28%] for surgical noncardiac cases). Hospital characteristics were similar across quartiles of site CPR duration (eTable 5 in Supplement 1).

**Table 1.** Hospital-Level Analysis: Characteristics of All Events Stratified by Hospital Quartile of Median CPR Duration in Events Without ROC

Characteristic	Median CPR duration among events without ROC, No./total No. (%) (N = 12 487)				*P* value
	Quartile 1 (15.0-25.9 min) (n = 2624)	Quartile 2 (26.0-29.4 min) (n = 4871)	Quartile 3 (29.5-32.9 min) (n = 2575)	Quartile 4 (33.0-53.0 min) (n = 2417)	
Year					
2000	18/94 (19)	31/94 (33)	17/94 (18)	28/94 (30)	<.001
2001	21/115 (18)	58/115 (50)	8/115 (7)	28/115 (24)	
2002	36/189 (19)	113/189 (60)	14/189 (7)	26/189 (14)	
2003	34/212 (16)	122/212 (58)	28/212 (13)	28/212 (13)	
2004	42/332 (13)	151/332 (45)	56/332 (17)	83/332 (25)	
2005	143/592 (24)	257/592 (43)	114/592 (19)	78/592 (13)	
2006	128/550 (23)	267/550 (49)	109/550 (20)	46/550 (8)	
2007	159/583 (27)	236/583 (40)	139/583 (24)	49/583 (8)	
2008	227/690 (33)	247/690 (36)	155/690 (22)	61/690 (9)	
2009	214/720 (30)	260/720 (36)	189/720 (26)	57/720 (8)	
2010	145/663 (22)	231/663 (35)	212/663 (32)	75/663 (11)	
2011	157/635 (25)	229/635 (36)	147/635 (23)	102/635 (16)	
2012	223/744 (30)	274/744 (37)	170/744 (23)	77/744 (10)	
2013	174/721 (24)	280/721 (39)	178/721 (25)	89/721 (12)	
2014	101/691 (15)	277/691 (40)	152/691 (22)	161/691 (23)	
2015	92/743 (12)	309/743 (42)	142/743 (19)	200/743 (27)	
2016	109/770 (14)	289/770 (38)	130/770 (17)	242/770 (31)	
2017	183/890 (21)	288/890 (32)	151/890 (17)	268/890 (30)	
2018	143/878 (16)	325/878 (37)	139/878 (16)	271/878 (31)	
2019	144/880 (16)	312/880 (35)	165/880 (19)	259/880 (29)	
2020	109/696 (16)	273/696 (39)	143/696 (21)	171/696 (25)	
2021	22/99 (22)	42/99 (42)	17/99 (17)	18/99 (18)	
Age group					
Neonates (0-30 d)	1125/4158 (27)	1368/4158 (33)	999/4158 (24)	666/4158 (16)	<.001
Infants (31 d to <1 y)	751/4163 (18)	1697/4163 (41)	831/4163 (20)	884/4163 (21)	
Young children (1-8 y)	341/2171 (16)	989/2171 (46)	389/2171 (18)	452/2171 (21)	
Older children (>8 y)	407/1995 (20)	817/1995 (41)	356/1995 (18)	415/1995 (21)	
Sex					
Female	1160/5531 (21)	2163/5531 (39)	1162/5531 (21)	1046/5531 (19)	.65
Male	1464/6956 (21)	2708/6956 (39)	1413/6956 (20)	1371/6956 (20)	
Race^a^					
Asian	30/377 (8)	131/377 (35)	76/377 (20)	140/377 (37)	.005
Black	938/3319 (28)	1321/3319 (40)	688/3319 (21)	372/3319 (11)	
White	1175/6345 (19)	2306/6345 (36)	1537/6345 (24)	1327/6345 (21)	
Other or unknown^b^	475/2396 (20)	1077/2396 (45)	274/2396 (11)	570/2396 (24)	
Hispanic ethnicity	530/2213 (24)	965/2213 (44)	279/2213 (13)	439/2213 (20)	<.001
Illness category^c^					
Medical cardiac	376/1858 (20)	808/1858 (43)	284/1858 (15)	390/1858 (21)	<.001
Medical noncardiac	967/5223 (19)	2382/5223 (46)	1050/5223 (20)	824/5223 (16)	
Surgical cardiac	284/1978 (14)	558/1978 (28)	453/1978 (23)	683/1978 (35)	
Surgical noncardiac	171/1115 (15)	437/1115 (39)	191/1115 (17)	316/1115 (28)	
Newborn	640/1654 (39)	396/1654 (24)	489/1654 (30)	129/1654 (8)	
Trauma	186/652 (29)	284/652 (44)	107/652 (16)	75/652 (12)	
Preexisting conditions					
Hypotension or hypoperfusion	925/3818 (24)	1385/3818 (36)	912/3818 (24)	596/3818 (16)	<.001
Respiratory insufficiency	1863/8463 (22)	3218/8463 (38)	1837/8463 (22)	1545/8463 (18)	<.001
Kidney insufficiency	271/1236 (22)	431/1236 (35)	281/1236 (23)	253/1236 (20)	.21
Sepsis^d^	576/1852 (31)	630/1852 (34)	383/1852 (21)	263/1852 (14)	<.001
Metastatic or hematologic malignant neoplasm	94/502 (19)	212/502 (42)	73/502 (15)	123/502 (25)	.15
Admission PCPC score^e^					
1	479/3454 (14)	1650/3454 (48)	663/3454 (19)	662/3454 (19)	<.001
2	142/1171 (12)	567/1171 (48)	268/1171 (23)	194/1171 (17)	
3	168/851 (20)	367/851 (43)	155/851 (18)	161/851 (19)	
≥4	354/1552 (23)	598/1552 (39)	314/1552 (20)	286/1552 (18)	
Pre-event interventions in place					
Mechanical ventilation	1878/9033 (21)	3533/9033 (39)	1846/9033 (20)	1776/9033 (20)	.25
Invasive airway	1321/5724 (23)	2325/5724 (41)	1158/5724 (20)	920/5724 (16)	<.001
Vasoactive agent	818/4203 (19)	1514/4203 (36)	903/4203 (21)	968/4203 (23)	<.001
Event location					
ICU	2095/9724 (22)	3702/9724 (38)	2029/9724 (21)	1898/9724 (20)	.93
Monitored	47/192 (24)	51/192 (27)	64/192 (33)	30/192 (16)	
Nonmonitored	85/508 (17)	217/508 (43)	100/508 (20)	106/508 (21)	
ED	177/836 (21)	425/836 (51)	145/836 (17)	89/836 (11)	
Procedural	101/869 (12)	385/869 (44)	127/869 (15)	256/869 (29)	
Other	119/358 (33)	91/358 (25)	110/358 (31)	38/358 (11)	
Initial rhythm					
Asystole	451/1842 (24)	669/1842 (36)	388/1842 (21)	334/1842 (18)	<.001
Bradycardia	1562/7277 (21)	2837/7277 (39)	1436/7277 (20)	1442/7277 (20)	
PEA	512/2694 (19)	1076/2694 (40)	638/2694 (24)	468/2694 (17)	
VF	51/336 (15)	141/336 (42)	53/336 (16)	91/336 (27)	
Pulseless VT	48/338 (14)	148/338 (44)	60/338 (18)	82/338 (24)	
Survival to hospital discharge	954/5339 (18)	2163/5339 (41)	1112/5339 (21)	1110/5339 (21)	<.001

Abbreviations: CPR, cardiopulmonary resuscitation; ED, emergency department; ICU, intensive care unit; PCPC, Pediatric Cerebral Performance Category; PEA, pulseless electrical activity; ROC, return of circulation; VF, ventricular fibrillation; VT, ventricular tachycardia.

^a^ Data were missing for 50 patients.

^b^ Includes self-reported American Indian or Alaska Native, Native Hawaiian or Other Pacific Islander, other, and unknown.

^c^ Data were missing for 7 patients.

^d^ Data were missing for 878 patients.

^e^ Data were missing for 5459 patients. Scores range from 1 (normal) to 6 (death).

Table 2 reports survival outcomes among all patients stratified by hospital median CPR duration. Patients at quartile 4 hospitals had a 10% absolute higher rate of survival to discharge (1110 of 2417 [46%]) compared with quartile 1 hospitals (954 of 2624 [36%]). After multivariable adjustment, patients who received CPR in hospitals with median CPR durations in quartiles 2 (adjusted odds ratio [AOR], 1.22 [95% CI, 1.09-1.36] *P* < .001) and 3 (AOR, 1.23 [95% CI, 1.08-1.39]; *P* = .002), but not quartile 4 (AOR, 1.04 [95% CI, 0.91-1.19]; *P* = .58), had higher odds of survival to hospital discharge compared with patients at hospitals in quartile 1. For the logistic regression analysis of survival to hospital discharge (primary outcome), C = 0.762 (Hosmer Lemeshow *P* = .43).

**Table 2.** Hospital-Level Analysis: Patient Outcomes Stratified by Hospital Quartile of Median CPR Duration in Events Without ROC

Outcome	Quartile 1 (15.0-25.9 min) (n = 2624)	Quartile 2 (26.0-29.4 min) (n = 4871)	Quartile 3 (29.5-32.9 min) (n = 2575)	Quartile 4 (33.0-53.0 min) (n = 2417)
Return of spontaneous circulation				
AOR (95% CI)	1 [Reference]	1.37 (1.22-1.54)	1.10 (0.97-1.25)	1.19 (1.03-1.37)
*P* value	NA	<.001	.15	.02
Survival to hospital discharge				
AOR (95% CI)	1 [Reference]	1.22 (1.09-1.36)	1.23 (1.08-1.39)	1.04 (0.91-1.19)
*P* value	NA	<.001	.002	.58

Abbreviations: AOR, adjusted odds ratio; CPR, cardiopulmonary resuscitation; NA, not applicable; ROC, return of circulation.

## Discussion

In this cohort study, we investigated pediatric IHCA CPR duration, identifying considerable variability in the duration of CPR provided to children without ROC among GWTG-R sites. In our hospital-level analysis, with sites stratified into quartiles based on median CPR duration calculated using events without ROC, we found that the adjusted odds of survival to discharge were lower for patients receiving CPR at hospitals that performed the shortest (quartile 1) and longest (quartile 4) median durations of CPR. In the patient-level analysis of events without ROC, male sex and Black race were not associated with CPR duration, though the variability was partially explained by neonatal age, initial rhythm, and event location.

In our analysis of outcomes stratified by hospital median CPR duration, we found that patients receiving CPR at hospitals with the shortest median CPR durations in events without ROC (quartile 1) had lower rates of survival to discharge than patients at hospitals in quartiles 2 and 3. We speculate that this could indicate that at quartile 1 hospitals, resuscitation efforts are aborted in some proportion of patients with the potential to survive. Previous pediatric literature^6^ has noted that IHCA survival rates exceed 10%, even among those receiving CPR for more than 35 minutes, and that certain patient characteristics, including illness category, are associated with better outcomes after prolonged CPR. Our findings suggest a need to explore interhospital variation in pediatric resuscitation practices, with the goal of avoiding premature termination of resuscitation among children with a reasonable likelihood of survival after in-hospital CPR.

Contrary to our hypothesis and adult data, patients at sites with the longest median CPR duration did not have higher odds of survival to discharge.^8^ We hypothesize that quartile 4 hospitals may be more likely to perform long CPR attempts in moribund patients unlikely to survive. A higher proportion of events at quartile 4 hospitals occurred in 2014 or later, when GWTG-R survival rates were higher.^2^ Additionally, a higher proportion of patients at quartile 4 hospitals were nonneonates, with previous studies demonstrating different risk-standardized survival rates across pediatric age groups.^12^ However, while patients at quartile 4 hospitals did not have a statistically significant higher adjusted odds of survival to discharge, they did have a higher likelihood of ROSC and a 10% absolute higher rate of survival to discharge compared with quartile 1 hospitals (46% vs 36%, respectively). We may have been unable to detect a difference due to variable event characteristics between quartile 4 hospitals compared with quartiles 1 to 3 hospitals that were controlled for in our analysis.

The variability among GWTG-R hospitals in CPR duration performed in events without ROC is substantial but unsurprising. In the absence of evidence defining optimal CPR duration, AHA life support guidelines do not provide specific recommendations for timing of termination of resuscitation.^18,19^ The complex decision of when to stop CPR is appropriately left to the clinical team. Previous work has demonstrated clinician-level differences in determining when to terminate resuscitation, including the impact of pediatric subspecialty and a wide breadth of factors that clinicians consider.^20,21^ Institutional practices also undoubtedly influence CPR duration. For example, clinicians at institutions with ECPR programs may be more accustomed to the longer resuscitations sometimes required to implement ECPR.^22,23^

Our analysis of factors associated with CPR duration in events without ROC highlighted several important findings. First, unlike in adult IHCA, patient sex was not associated with CPR duration.^7^ This may be because several of the proposed mechanisms for sex-based differences in cardiac arrest interventions and outcomes are less applicable to children.^24^ Second, neonates, who comprised 34% of our cohort, received shorter durations of CPR compared with older children. Interpreting this association requires understanding not only of the patient population but also differences in care settings. Conditions specific to these patients, namely, life-limiting congenital malformations and considerations related to prematurity and/or viability, may contribute to differences in prognostication and resuscitative effort duration. Moreover, most neonatal events in our cohort (67%) occurred in NICUs. Resuscitation practices likely differ between clinical locations, which is supported by our finding that event location was associated with CPR duration and previous investigations of infant resuscitation.^25,26,27^ Third, patients with initial rhythms of bradycardia with poor perfusion and PEA received longer durations of CPR than those with an initial rhythm of asystole, which may be related to clinician perception of a higher likelihood of survival in patients with these rhythms.^4,28^

With pervasive disparities affecting vulnerable children,^29^ it was important for our study to determine whether there was an association between race and CPR duration among event nonsurvivors. On multivariable analysis, we did not find that Black or Asian children without ROC received a different CPR duration compared with White children. However, in the hospital-level analysis, race was associated with hospital median CPR duration in events without ROC. A hospital’s tendency to perform shorter or longer durations of CPR may be accompanied by additional resuscitation practices not measured in our study that impact outcomes. In fact, hospital differences explain much of the association between Black race and worse adult IHCA outcomes.^30,31^ While previous GWTG-R analysis has shown that pediatric IHCA survival outcomes do not differ based on race,^30^ our finding that race is associated with site median CPR duration supports the need for investigation of hospital pediatric resuscitation practices and patient racial composition.

### Limitations

This study should be interpreted in light of several limitations. Duration of CPR among events without ROC is an imperfect proxy for resuscitation effort intensity. Generalizability is limited by the registry’s voluntary nature, with possible differences between participating and nonparticipating hospitals. We cannot confirm that participating hospitals entered all events into the registry. At low-volume sites, site median CPR duration among events without ROC was derived from a small number of events and may not represent hospital CPR duration practices more broadly. Clinician and family decision-making related to termination of resuscitation could not be assessed. For characteristics associated with CPR duration, the mechanism cannot be elucidated. Survival to discharge and ROC do not equate to favorable neurologic outcomes; however, we could not evaluate neurologic outcome due to high missingness of discharge PCPC.

There are several unmeasured potential confounders, including hospital resuscitation processes and complexity of comorbidities (eg, prematurity), which are described in limited detail in the dataset and could play an important role in both CPR duration and outcomes. In addition, while ECPR events did not contribute to site median CPR duration, having an ECPR program may still predispose sites to performing longer resuscitations. The years included in our study also correspond temporally with increased pediatric IHCA prevention efforts, including rapid response system development and situational awareness efforts, which are not captured in our dataset but could have changed IHCA characteristics and hospital resuscitation practices. Our study did not address combinations of factors that may contribute to decisions regarding timing of termination of resuscitation, nor did we explore the issue of possible collinearity between variables.

## Conclusions

In this multicenter, retrospective cohort study of cardiac arrest, factors including age, event location, and initial rhythm, but not sex or Black race, were associated with CPR duration among children without ROC. There was substantial interhospital variability in the duration of CPR attempts during events without ROC. The odds of survival to discharge were lower for patients at hospitals in the shortest and longest quartiles of median CPR duration among events without ROC. Further investigation is needed to determine the optimal CPR duration during pediatric IHCA and to provide training guidelines for resuscitation teams to eliminate disparities in resuscitation care.

## Notes

Supplement 1. **eTable 1.** List of Variables Included in the Model **eTable 2.** Patient-Level Analysis: Characteristics of Events Without Return of Circulation Stratified by Quartile of CPR Duration **eTable 3.** Patient Characteristics: All Eligible Events by Resuscitation Status **eFigure 1.** Distribution of CPR Duration in All Events **eFigure 2.** Distribution of CPR Duration in Events With Return of Circulation **eTable 4.** Multivariable Model of Patient-Level Analysis: Factors Associated With CPR Duration Among Events Without Return of Circulation **eFigure 3.** Unabbreviated Forest Plot of Patient-Level Analysis Multivariable Model: Patient and Event Factors Associated With CPR Duration in Events Without Return of Circulation **eTable 5.** Hospital-Level Analysis: Site Characteristics by Hospital Quartile of Median CPR Duration in Events Without Return of Circulation

Supplement 2.
Nonauthor Collaborators

Supplement 3.
Data Sharing Statement
