# Error in Abstract

**DOI:** 10.1001/jamanetworkopen.2024.35956

**Published:** 2024-08-28

In the Original Investigation titled “Characteristics of Pediatric In-Hospital Cardiac Arrests and Resuscitation Duration,”^1^ published July 30, 2024, the number 7 was inadvertently inserted within the word *care* in the Results section of the abstract. This article has been corrected.
