# Supplementary material for: Characteristics of Pediatric In-Hospital Cardiac Arrests and Resuscitation Duration
Source: JAMA Netw Open. 2024 Jul 30;7(7):e2424670. doi: 10.1001/jamanetworkopen.2024.24670 (PMC11289702; doi:10.1001/jamanetworkopen.2024.24670)
Supplement: Supplement 1. — eTable 1. List of Variables Included in the Model eTable 2. Patient-Level Analysis: Characteristics of Events Without Return of Circulation Stratified by Quartile of CPR Duration eTable 3. Patient Characteristics: All Eligible Events by Resuscitation Status eFigure 1. Distribution of CPR Duration in All Events eFigure 2. Distribution of CPR Duration in Events With Return of Circulation eTable 4. Multivariable Model of Patient-Level Analysis: Factors Associated With CPR Duration Among Events Without Return of Circulation eFigure 3. Unabbreviated Forest Plot of Patient-Level Analysis Multivariable Model: Patient and Event Factors Associated With CPR Duration in Events Without Return of Circulation eTable 5. Hospital-Level Analysis: Site Characteristics by Hospital Quartile of Median CPR Duration in Events Without Return of Circulation [file jamanetwopen-e2424670-s001.pdf]

Supplementary Online Content

O'Halloran A, Morgan RW, Kennedy K, et al; American Heart Association's Get With the Guidelines–Resuscitation Investigators. Characteristics of pediatric in-hospital cardiac arrests and resuscitation duration. *JAMA Netw Open*. 2024;7(7):e2424670. doi:10.1001/jamanetworkopen.2024.24670

eTable 1. List of Variables Included in the Model

eTable 2. Patient-Level Analysis: Characteristics of Events Without Return of Circulation Stratified by Quartile of CPR Duration

eTable 3. Patient Characteristics: All Eligible Events by Resuscitation Status

eFigure 1. Distribution of CPR Duration in All Events

eFigure 2. Distribution of CPR Duration in Events With Return of Circulation

eTable 4. Multivariable Model of Patient-Level Analysis: Factors Associated With CPR Duration Among Events Without Return of Circulation

eFigure 3. Unabbreviated Forest Plot of Patient-Level Analysis Multivariable Model: Patient and Event Factors Associated With CPR Duration in Events Without Return of Circulation

eTable 5. Hospital-Level Analysis: Site Characteristics by Hospital Quartile of Median CPR Duration in Events Without Return of Circulation

This supplementary material has been provided by the authors to give readers additional information about their work.

© 2024 O'Halloran A et al. *JAMA Network Open*.

eTable 1. List of Variables Included in the Model

Demographics
Calendar year
Age at the time of cardiac arrest, divided into four groups<br>Neonates (≤30 days), infants (31 days to <1 year), young children (1-8 years), and older children (>8 years of age)
Sex
Race
Illness category, categorized as medical cardiac, medical noncardiac, surgical cardiac, surgical noncardiac, trauma, and newborn
Pre-existing conditions
Congestive heart failure (this admission)
Congestive heart failure (prior to this admission)
Hypotension/hypoperfusion
Renal insufficiency
Hepatic insufficiency
Metabolic/electrolyte abnormality
Pneumonia
Major trauma
Interventions Already in Place
Mechanical ventilation
Vasoactive agent
Event Characteristics
Hospital-wide alert
Automated external defibrillator applied
Initial rhythm, categorized as ventricular fibrillation, pulseless ventricular tachycardia, pulseless electrical activity, asystole, and bradycardia with poor perfusion
Time of arrest, day vs night
Event location, categorized as pediatric intensive care unit, emergency department, neonatal intensive care unit, cardiac intensive care unit, other intensive care unit, operating room or procedural area, newborn area, and other inpatient area

© 2024 O'Halloran A et al. *JAMA Network Open*.

eTable 2. Patient-Level Analysis: Characteristics of Events Without Return of Circulation Stratified by Quartile of CPR Duration

	Total n=3859	Event Duration (minutes) Quartile 1 (0 to 15 min) n=897	Quartile 2 (16 to 27 min) n=963	Quartile 3 (28 to 45 min) n=1022	Quartile 4 (46 to 300 min) n=977	P-value
**Demographics and Patient Characteristics**						
**Event Year**						.66
2000	48	6/48 (13%)	10/48 (21%)	15/48 (31%)	17/48 (35%)	
2001	60	10/60 (17%)	11/60 (18%)	19/60 (32%)	20/60 (33%)	
2002	103	18/103 (17%)	26/103 (25%)	33/103 (30%)	26/103 (25%)	
2003	113	24/113 (21%)	31/113 (27%)	27/113 (24%)	31/113 (27%)	
2004	149	30/149 (20%)	33/149 (22%)	39/149 (26%)	47/149 (32%)	
2005	225	58/225 (26%)	57/225 (25%)	52/225 (23%)	58/225 (26%)	
2006	203	50/203 (25%)	62/203 (31%)	50/203 (25%)	41/203 (20%)	
2007	210	60/210 (29%)	52/210 (25%)	42/210 (20%)	56/210 (27%)	
2008	215	43/215 (20%)	67/215 (31%)	51/215 (24%)	54/215 (25%)	
2009	228	48/228 (21%)	64/228 (28%)	59/228 (26%)	57/228 (25%)	
2010	192	50/192 (26%)	46/192 (24%)	52/192 (27%)	44/192 (23%)	
2011	170	43/170 (25%)	43/170 (25%)	44/170 (26%)	40/170 (24%)	
2012	238	58/238 (22%)	57/238 (24%)	60/238 (25%)	63/238 (26%)	
2013	194	55/194 (28%)	46/194 (24%)	53/194 (27%)	40/194 (21%)	
2014	188	35/188 (19%)	43/188 (23%)	52/188 (28%)	58/188 (31%)	
2015	190	52/190 (27%)	59/190 (31%)	45/190 (24%)	34/190 (18%)	
2016	190	37/190 (19%)	45/190 (24%)	63/190 (33%)	45/190 (24%)	
2017	268	63/268 (24%)	61/268 (23%)	76/268 (28%)	68/268 (25%)	
2018	220	58/220 (26%)	51/220 (23%)	55/220 (25%)	56/220 (25%)	
2019	227	45/227 (20%)	54/227 (24%)	65/227 (29%)	63/227 (28%)	
2020	203	47/203 (23%)	39/203 (19%)	63/203 (31%)	54/203 (27%)	
2021	25	7/25 (28%)	6/25 (24%)	7/25 (28%)	5/25 (20%)	
**Age Groups**						.002
Neonates (0-30 days)	1315	370/1315 (28%)	345/1315 (26%)	324 (25%)	276 (21%)	
Infants (31 days-1 year)	927	169/927 (18%)	214/927 (23%)	281/927 (30%)	263/927 (28%)	
Young children (1-8 years)	746	135/746 (18%)	190/746 (25%)	212/746 (28%)	209/746 (28%)	
Older children (>8 years)	871	223/871 (26%)	214/871 (25%)	205/871 (24%)	229/871 (26%)	
**Sex**						.91
Female	1684	387/1684 (23%)	422/1684 (25%)	452/1684 (27%)	423/1684 (25%)	
Male	2175	510/2175 (23%)	541/2175 (25%)	570/2175 (26%)	554/2175 (25%)	
**Race**						0.002
Asian	106	23/106 (22%)	14/106 (13%)	37/106 (35%)	32/106 (30%)	
Black	1070	252/1070 (24%)	263/1070 (25%)	282/1070 (26%)	273/1070 (26%)	
Other/Unknown	795	207/795 (26%)	219/795 (28%)	198/795 (25%)	171/795 (22%)	
White	1888	415/1888 (22%)	467/1888 (25%)	505/1888 (27%)	501/1888 (27%)	
**Ethnicity**						
Hispanic	705	171/705 (24%)	196/705 (28%)	172/705 (24%)	166/705 (24%)	0.08
**Illness Category**						<0.001
Medical Cardiac	601	113/601 (19%)	154/601 (26%)	171/601 (28%)	163/601 (27%)	
Medical Noncardiac	1750	392/1750 (22%)	431/1750 (25%)	475/1750 (27%)	452/1750 (26%)	
Surgical Cardiac	322	45/322 (14%)	73/322 (23%)	99/322 (31%)	105/322 (33%)	
Surgical Noncardiac	228	45/228 (20%)	44/228 (19%)	69/228 (30%)	70/228 (31%)	
Newborn	584	171/584 (29%)	156/584 (27%)	136/584 (23%)	121/584 (21%)	
Trauma	374	131/374 (35%)	105/374 (28%)	72/374 (19%)	66/374 (18%)	
**Pre-Existing Conditions**						
Acyanotic Cardiac Malformation[a]	262	39/262 (15%)	69/262 (26%)	90/262 (34%)	64/262 (24%)	.02
Cyanotic Cardiac Malformation [a]	374	65/374 (17%)	77/374 (21%)	120/374 (21%)	112/374 (30%)	<.001

© 2024 O'Halloran A et al. *JAMA Network Open*.

Noncardiac Congenital Malformation [a]	457	94/457 (21%)	111/457 (24%)	139/457 (30%)	113/457 (25%)	.17
Hypotension/hypoperfusion	1392	394/1392 (28%)	355/1392 (26%)	336/1392 (24%)	307/1392 (22%)	<.001
Respiratory insufficiency	2426	594/2426 (24%)	591/2426 (24%)	631/2426 (26%)	610/2426 (25%)	.13
Renal insufficiency	453	137/453 (30%)	116/453 (26%)	100/453 (22%)	100/453 (22%)	<.001
Sepsis[b]	727	176/727 (24%)	172/727 (24%)	187/727 (26%)	192/727 (26%)	.96
Metastatic/hematologic mali gnancy	236	77/236 (%)	52/236 (22%)	47/236 (20%)	60/236 (25%)	.02
**Admission Pediatric Cerebral Performance Category**[c]						<0.001
1	948	169/948 (18%)	202/948 (21%)	255/948 (27%)	322/948 (34%)	
2	315	50/315 (16%)	69/315 (22%)	106/315 (34%)	90/315 (29%)	
3	248	62/248 (25%)	58/248 (23%)	67/248 (27%)	61/248 (25%)	
4+	704	204/704 (29%)	182/704 (26%)	170/704 (24%)	148/704 (21%)	
**Event Characteristics**						
**Pre-Event Interventions in Place**						
Mechanical ventilation	2843	767/2843 (27%)	728/2843 (26%)	688/2843 (24%)	660/2843 (23%)	<.001
Invasive airway	1869	507/1869 (27%)	508/1869 (27%)	431/1869 (23%)	423/1869 (23%)	<.001
Vasoactive agent	1578	471/1578 (30%)	409/1578 (26%)	350/1578 (22%)	348/1578 (22%)	<.001
**Event Location**						0.20
ICU	2809	713/2809 (25%)	670/2809 (24%)	724/2809 (26%)	702/2809 (25%)	
Monitored	62	5/62 (8%)	11/62 (18%)	22/62 (35%)	24/62 (39%)	
Non-Monitored	150	12/150 (8%)	40/150 (27%)	45/150 (30%)	53/150 (15%)	
Emergency Department	587	111/587 (19%)	164/587 (28%)	174/587 (30%)	138/587 (24%)	
Procedural	163	31/163 (19%)	43/163 (26%)	42/163 (26%)	47/163 (29%)	
Other	88	25/88 (28%)	35/88 (40%)	15/88 (17%)	13/88 (15%)	
**Initial Rhythm**						.001
Asystole	963	232/963 (24%)	282/963 (29%)	279/963 (29%)	170/963 (18%)	
Bradycardia	1711	393/1711 (23%)	398/1711 (23%)	464/1711 (27%)	456/1711 (27%)	
PEA	984	225/984 (23%)	230/984 (23%)	238/984 (24%)	291/984 (30%)	
VF	94	23/94 (24%)	26/94 (28%)	23/94 (24%)	22/94 (23%)	
Pulseless VT	107	24/107 (22%)	27/107 (25%)	18/107 (17%)	38/107 (36%)	

[a]928 missing; [b]220 missing; [c]1644 missing; The race Other/Unknown category includes self-reported registry data from the following groupings: American Indian or Alaska Native, Native Hawaiian or Other Pacific Islander, other, and unknown; CPR: cardiopulmonary resuscitation; ICU: intensive care unit; PEA: pulseless electrical activity; VF: ventricular fibrillation; VT: ventricular tachycardia

© 2024 O'Halloran A et al. *JAMA Network Open*.

eTable 3. Patient Characteristics: All Eligible Events by Resuscitation Status

	**Total** (n=13,899)	**ROC** (n=10,040)	**No ROC** (n=3,859)	**P-value**
**Demographics and Patient Characteristics**	No	No/Total No (%)	No/Total No (%)	
**Event Year**				<.001
2000	104	56/104 (54%)	48/104 (46%)	
2001	162	102/162 (63%)	60/162 (37%)	
2002	246	143/246 (58%)	103/246 (42%)	
2003	285	172/285 (60%)	113/285 (40%)	
2004	399	250/399 (63%)	149/399 (37%)	
2005	720	495/720 (69%)	225/720 (31%)	
2006	654	451/654 (69%)	203/654 (31%)	
2007	680	470/680 (69%)	210/680 (31%)	
2008	760	545/760 (72%)	215/760 (28%)	
2009	799	571/799 (72%)	228/799 (29%)	
2010	715	523/715 (73%)	192/715 (27%)	
2011	675	505/675 (75%)	170/675 (25%)	
2012	813	575/813 (71%)	238/813 (29%)	
2013	776	582/776 (75%)	194/776 (25%)	
2014	736	548/736 (75%)	188/736 (26%)	
2015	784	594/784 (76%)	190/784 (24%)	
2016	822	632/822 (77%)	190/822 (23%)	
2017	972	704/972 (72%)	268/972 (28%)	
2018	979	759/979 (78%)	220/979 (23%)	
2019	937	710/937 (76%)	227/937 (24%)	
2020	770	567/770 (74%)	203/770 (26%)	
2021	111	86/111 (78%)	25/111 (23%)	
**Age Groups**				<.001
Neonates (0-30 days)	4513	3198/4513 (71%)	1315 (29%)	
Infants (31 days-1 year)	4594	3667/4594 (80%)	927/4594 (20%)	
Young children (1-8 years)	2406	1660/2406 (69%)	746/2406 (31%)	
Older children (>8 years)	2386	1515/2386 (63%)	871/2386 (37%)	
**Race**				0.015
Asian	411	305/411 (74%)	106/411 (26%)	
Black	3633	2563/3633 (71%)	1070/3633 (29%)	
Other/Unknown	2741	1946/2741 (71%)	795/2741 (29%)	
White	7114	5226/7114 (73%)	1888/7114 (27%)	
**Illness Category**[a]				<.001
Medical Cardiac	2100	1499/2100 (71%)	601/2100 (29%)	
Medical Noncardiac	5816	4066/5816 (70%)	1750/5816 (30%)	
Surgical Cardiac	2013	1691/2013 (84%)	322/2013 (16%)	
Surgical Noncardiac	1183	955/1183 (81%)	228/1183 (19%)	
Newborn	1927	1343/1927 (70%)	584/1927 (30%)	
Trauma	853	479/853 (56%)	374/853 (44%)	
**Pre-Existing Conditions**				
Hypotension/hypoperfusion	4097	2705/4097 (66%)	1392/4097 (34%)	<.001
Respiratory insufficiency	9101	6675/9101 (73%)	2426/9101 (27%)	<.001
Renal insufficiency	1330	877/1330 (66%)	453/1330 (34%)	<.001
Sepsis	1986	1259/1986 (63%)	727/1986 (37%)	<.001
Metastatic/hematologic malignancy	534	298/534 (56%)	236/534 (44%)	<.001
**Event Characteristics**				
**Pre-Arrest Interventions**				
Assisted/mechanical ventilation	10306	7377/10306 (72%)	2929/10306 (28%)	.003
Invasive airway	6187	4318/6187 (70%)	1869/6187 (30%)	<.001
Vasoactive agent	4481	2903/4481 (65%)	1578/4481 (35%)	<.001
**Initial Rhythm**				<.001
Asystole	2276	1313/2276 (58%)	963/2276 (42%)	
Bradycardia with poor perfusion	7820	6109/7820 (78%)	1711/7820 (22%)	

© 2024 O’Halloran A et al. *JAMA Network Open*.

PEA	3014	2030/3014 (67%)	984/3014 (33%)	
VF	397	303/397 (76%)	94/397 (24%)	
Pulseless VT	392	285/392 (73%)	107/392 (27%)	

[a]7 missing; The race Other/Unknown category includes self-reported registry data from the following groupings: American Indian or Alaska Native, Native Hawaiian or Other Pacific Islander, other, and unknown; Sex, Hispanic ethnicity, and event location were not significantly associated with CPR duration and thus, have been removed from this table. ROC: return of circulation; ICU: intensive care unit; PEA: pulseless electrical activity; VF: ventricular fibrillation; VT: ventricular tachycardia

© 2024 O'Halloran A et al. *JAMA Network Open*.

eFigure 1. Distribution of CPR Duration in All Events

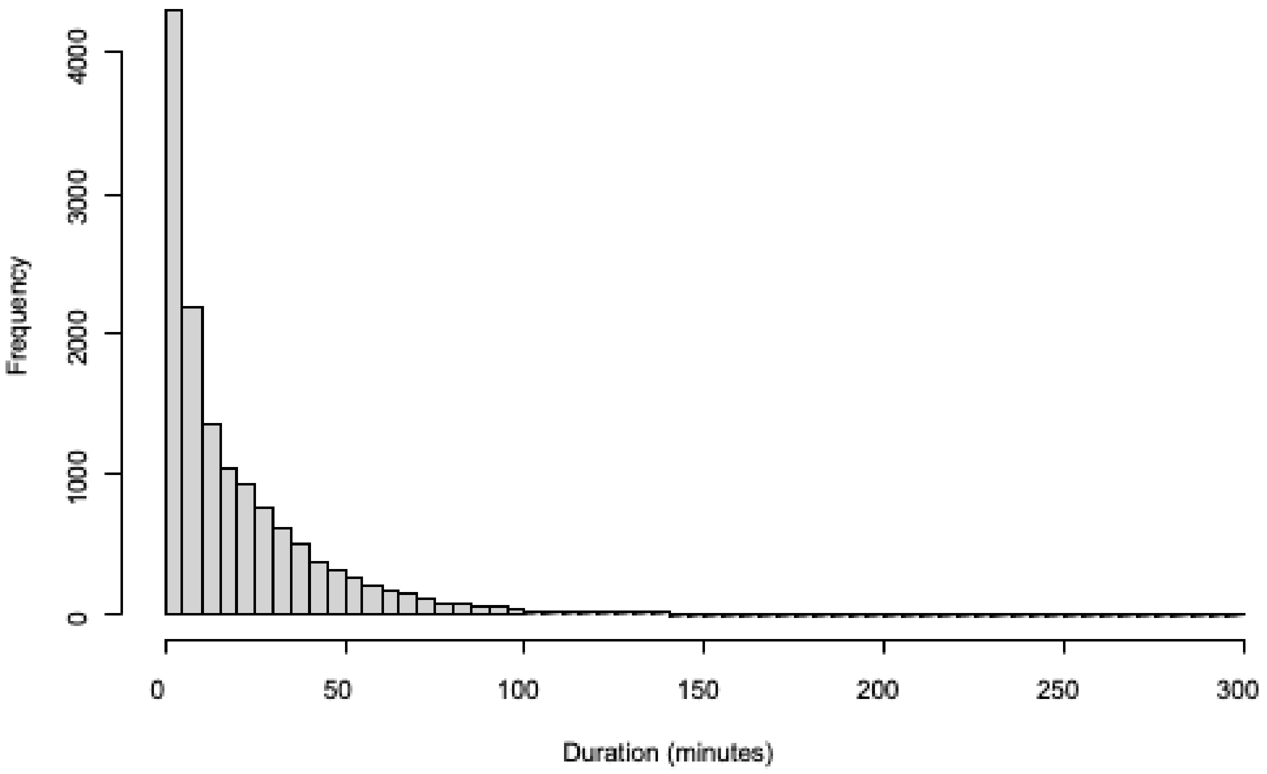

eFigure 2. Distribution of CPR Duration in Events With Return of Circulation

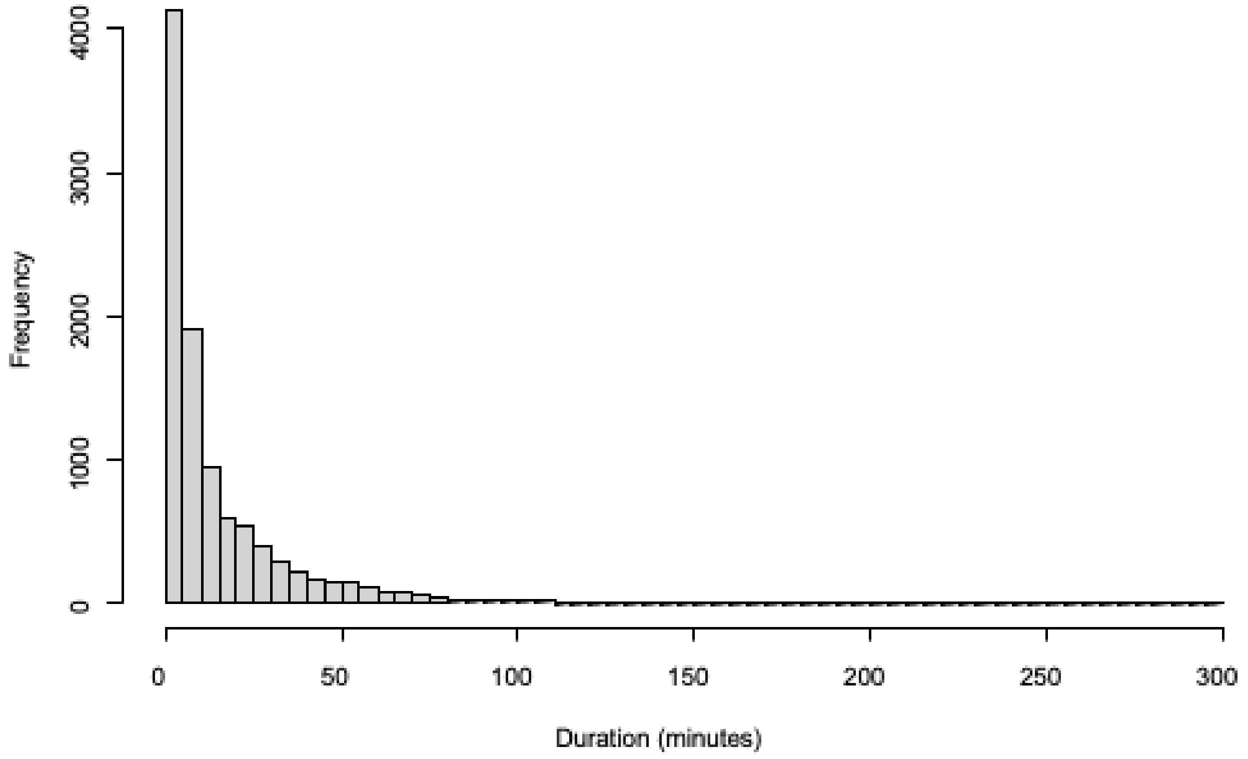

© 2024 O'Halloran A et al. *JAMA Network Open*.

eTable 4. Multivariable Model of Patient-Level Analysis: Factors Associated With CPR Duration Among Events Without Return of Circulation

**Factor**	**Beta Weight predicting duration** (minutes, 95% CI)	**P-value**
Year +1	-0.14 (-0.37, 0.08)	.21
**Age** (reference: older children)		
Neonate	-4.86 (-8.88, -0.84)	.02
Infant	0.26 (-2.78, 3.29)	.87
Young children	0.02 (-2.97, 3.02)	.99
**Sex**: Male vs female	1.19 (-0.72, 3.1)	.22
**Race** (reference: white)		
Black	0.65 (-1.61, 2.9)	.57
Asian	3.56 (-2.29, 9.42)	.23
Other/unknown	-3.67 (-6.18, -1.17)	.004
**Initial Rhythm** (reference: asystole)		
Bradycardia with poor perfusion	8.37 (5.7, 11.03)	<.0001
PEA	8.22 (5.44, 11)	<.0001
VF	-0.17 (-6.56, 6.22)	.96
Pulseless VT	6.17 (0.09, 12.26)	.05
**Event Location** (reference: PICU)		
ED	-4.02 (-7.48, -0.57)	.02
NICU	-3.74 (-7.74, 0.25)	.07
Pediatric CICU	2.97 (-1.86, 7.79)	.23
Other ICU	-0.24 (-3.87, 3.39)	.90
Operative/Procedural Area	3.15 (-2.08, 8.37)	.24
Newborn Area	-6.86 (-14.21, 0.49)	.07
Other inpatient area	1.06 (-4.1, 6.22)	.69
**Illness Category** (reference: medical cardiac)		
Medical non-cardiac	0.74 (-2.25, 3.72)	.63
Surgical cardiac	4.02 (-0.3, 8.35)	.07
Surgical non-cardiac	8.33 (3.47, 13.19)	<.001
Newborn	1.63 (-2.51 ,5.78)	.44
Trauma	-4.8 (-11.53, 1.92)	.16
**Pre-Existing Conditions**		
Heart failure this admission	4.51 (-0.1, 9.12)	.06
Heart failure prior to this admission	-4.64 (-9.7, 0.41)	.07
Hypotension	-0.83 (-3.03, 1.38)	.46
Renal insufficiency	-4.38 (-7.57, -1.2)	.007
Hepatic insufficiency	-1.78 (-6.13, 2.57)	.42
Metabolic abnormality	-1.81 (-4.33, 0.71)	.16
Pneumonia	3.59 (-0.32, 7.5)	.07
Major trauma	-2.42 (-8.49, 3.64)	.43
**Pre-Event Interventions in Place**		
Mechanical ventilation	-7.6 (-10.34, -4.86)	<.0001
Vasoactive agent	-1.74 (-3.97, 0.49)	.13
Hospital-wide response activation	2.16 (-0.29, 4.62)	.08
AED applied	4.43 (1.39, 7.48)	.004

PEA: pulseless electrical activity; VF: ventricular fibrillation; VT: ventricular tachycardia; ED: emergency department; NICU: neonatal intensive care unit; CICU: cardiac intensive care unit; ICU: intensive care unit; AED: automated external defibrillator

© 2024 O’Halloran A et al. *JAMA Network Open*.

eFigure 3. Unabbreviated Forest Plot of Patient-Level Analysis Multivariable Model: Patient and Event Factors Associated With CPR Duration in Events Without Return of Circulation

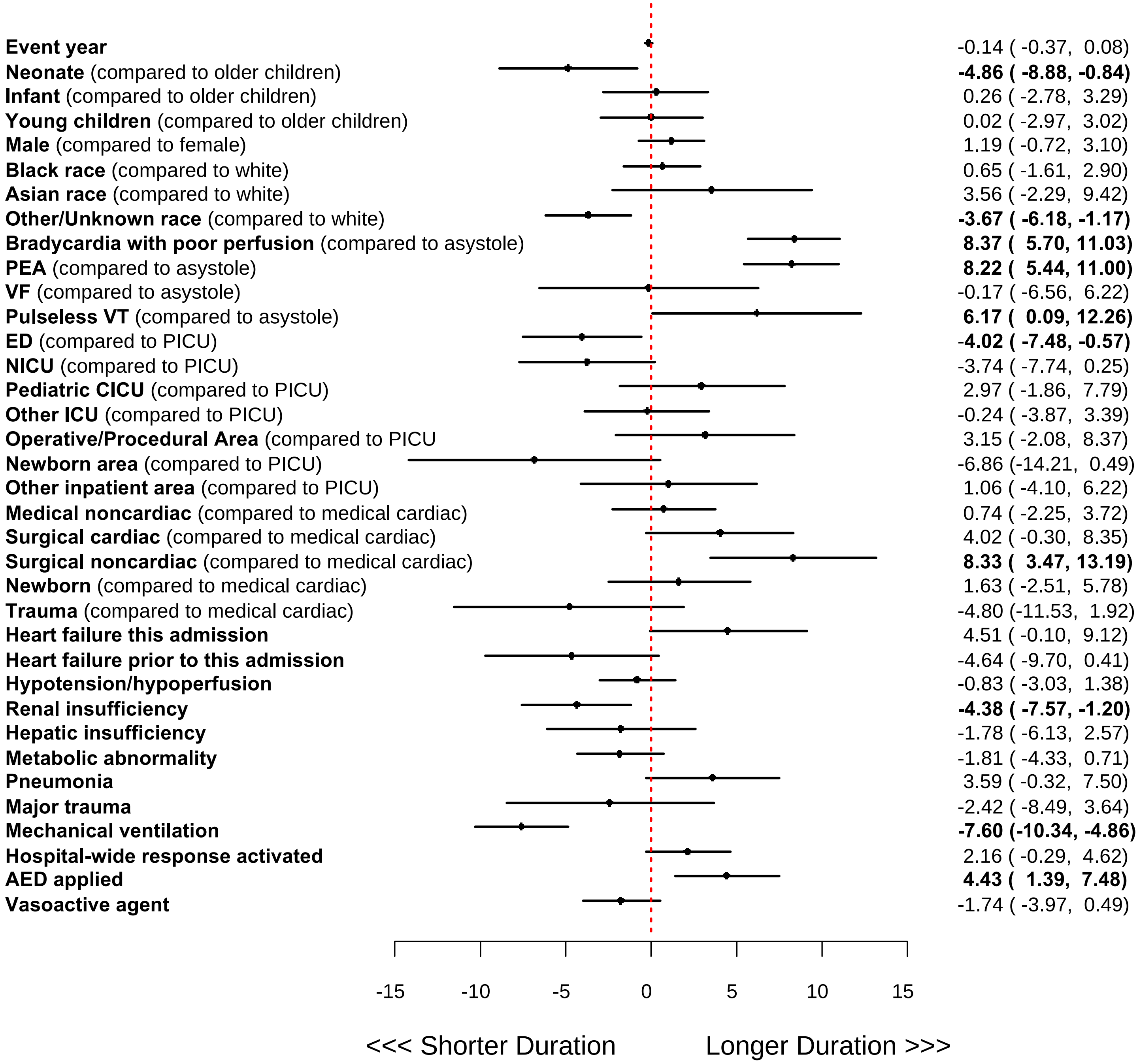

Patient and event factors are listed on the left. CPR duration in minutes (95% CI) is listed on the right.
PEA: pulseless electrical activity; VF: ventricular fibrillation; VT: ventricular tachycardia; ED: Emergency Department; NICU: Neonatal intensive care unit; CICU: Cardiac intensive care unit; ICU: intensive care unit; AED: automated external defibrillator

© 2024 O'Halloran A et al. *JAMA Network Open*.

eTable 5. Hospital-Level Analysis: Site Characteristics by Hospital Quartile of Median CPR Duration in Events Without Return of Circulation

Hospital Characteristic	All Sites n=81	Quartile of Median Event Duration				P-value
		**Quartile 1** (15 to 25.9 min) n=20	**Quartile 2** (26 to 29.4 min) n=20	**Quartile 3** (29.5 to 32.9 min) n=19	**Quartile 4** (22 to 53 min) n=22	
**Teaching Status**[a]						.24
None	3	0/3 (0%)	1/3 (33%)	2/3 (67%)	0/3 (0%)	
Minor	40	12/40 (30%)	7/40 (18%)	11/40 (28%)	10/40 (25%)	
Major	33	7/33 (21%)	11/33 (33%)	5/33 (15%)	10/33 (30%)	
Missing	5	1	1	1	2	
**Hospital Size (beds)**[a]						.07
<200	4	0/4 (0%)	3/4 (75%)	1/4 (25%)	0/4 (0%)	
200-499	30	7/30 (23%)	10/30 (33%)	4/30 (13%)	9/30 (30%)	
500+	42	12/42 (29%)	6/42 (14%)	13/42 (31%)	11/42 (26%)	
Missing	5	1	1	1	2	
**Census Division Region**[a]						.78
North MidAtlantic	13	3/13 (23%)	2/13 (15%)	4/13 (31%)	4/13 (31%)	
South Atlantic	22	8/22 (36%)	4/22 (18%)	6/22 (27%)	4/22 (18%)	
North Central	14	2/14 (14%)	4/14 (29%)	4/14 (29%)	4/14 (29%)	
South Central	15	5/15 (33%)	4/15 (27%)	2/15 (13%)	4/15 (27%)	
Mountain/Pacific	12	1/12 (8.3%)	5/12 (42%)	2/12 (17%)	4/12 (33%)	
Missing	5	1	1	1	2	

[a]5 missing
CPR: cardiopulmonary resuscitation; min: minutes

© 2024 O’Halloran A et al. *JAMA Network Open*.
