# Supplementary material for: Characteristics of Pediatric In-Hospital Cardiac Arrests and Resuscitation Duration
Source: JAMA Netw Open. 2024 Jul 30;7(7):e2424670. doi: 10.1001/jamanetworkopen.2024.24670 (PMC11289702; doi:10.1001/jamanetworkopen.2024.24670)
Supplement: Supplement 2. — Nonauthor Collaborators [file jamanetwopen-e2424670-s002.pdf]

Supplemental Online Content: Nonauthor Collaborators
*First name, last name, and suffix (if applicable) are required and will appear in PubMed.

*Group Name(s): the American Heart Association's Get With the Guidelines–Resuscitation Investigators							
***First Name and Middle Initial(s)**	***Last Name**	***Suffix (eg, Jr, III)**	Academic Degrees	Institution	Location (city, state/province, country)	Role or Contribution, eg, chair, principal investigator	Group (if more than 1 Group listed in the byline) and/or Subgroup (eg, Steering Committee)
Anne-Marie	Guerguerian		MD PhD FRCPC FAAP FAHA	Department of Critical Care Medicine, SickKids Research Institute, The Hospital for Sick Children, University of Toronto	Toronto, Ontario, Canada		
Caitlin E.	O’Brien		MD MPH	Johns Hopkins University School of Medicine, Department of Anesthesiology and Critical Care Medicine	Baltimore, Maryland, USA		
Ericka L.	Fink		MD MS	Children's Hospital of Pittsburgh of UPMC, Department of Critical Care Medicine	Pittsburgh, Pennsylvania, USA		
Javier J.	Lasa		MD FAAP	Children's Medical Center, UT Southwestern Medical Center	Dallas, Texas, USA		
Joan S.	Roberts		MD	Seattle Children's Hospital, University of Washington	Seattle, Washington, USA		
Lililan	Su		MD	Division of Cardiac Intensive Care, Department of Pediatrics, Phoenix Children's Hospital	Phoenix, Arizona, USA		
Linda L.	Brown		MD MSCE	The Warren Alpert Medical School of Brown University	Providence, Rhode Island, USA		
Maya	Dewan		MD MPH	Cincinnati Children's Hospital Medical Center; Department of Pediatrics, College of Medicine, University of Cincinnati; Division of Biomedical Informatics, Cincinnati Children's Hospital Medical Center	Cincinnati, Ohio, USA		
Monica	Kleinman		MD	Department of Anesthesiology, Critical Care and Pain Medicine, Boston Children's Hospital	Boston, Massachusetts, USA		

Supplemental Online Content: Nonauthor Collaborators
*First name, last name, and suffix (if applicable) are required and will appear in PubMed.

***First Name and Middle Initial(s)**	***Last Name**	***Suffix (eg, Jr, III)**	Academic Degrees	Institution	Location (city, state/province, country)	Role or Contribution, eg, chair, principal investigator	Group (if more than 1 Group listed in the byline) and/or Subgroup (eg, Steering Committee)
Noorjahan	Ali		MD MS FAAP	Division of Neonatal-Perinatal Medicine, Department of Pediatrics, University of Texas Southwestern	Dallas, Texas, USA		
Punkaj	Gupta		MBBS	Section of Cardiac Critical Care, Methodist Children's Hospital	San Antonio, Texas, USA		
Robert	Sutton		MD MSCE	Department of Anesthesia and Critical Care, The Children's	Philadelphia, Pennsylvania, USA		
Ron	Reeder		MS PhD	University of Utah School of Medicine	Salt Lake City, Utah, USA		
Todd	Sweberg		MD MBA	Department of Pediatric Critical Care, Cohen Children's Medical Center of New York	New Hyde Park, New York, USA		
