# Supplementary material for: Characteristics of Pediatric In-Hospital Cardiac Arrests and Resuscitation Duration
Source: JAMA Netw Open. 2024 Jul 30;7(7):e2424670. doi: 10.1001/jamanetworkopen.2024.24670 (PMC11289702; doi:10.1001/jamanetworkopen.2024.24670)
Supplement: Supplement 3. — Data Sharing Statement [file jamanetwopen-e2424670-s003.pdf]

Data Sharing Statement

O'Halloran. Characteristics of Pediatric In-Hospital Cardiac Arrests and Resuscitation Duration. *JAMA Netw Open*. Published July 30, 2024. doi:10.1001/jamanetworkopen.2024.24670

Data
Data available: No

Additional Information
Explanation for why data not available: The data that support the findings of this study are available from the American Heart Association's Get With The Guidelines Registry. The data, which are collected primarily for quality improvement purposes, are available through the American Heart Association's application process.
